# Genomic Profiling of Breast Cancer in an Ecuadorian Cohort Reveals Clinically Relevant Variants and Ancestry-Related Interpretation Challenges

**DOI:** 10.3390/cancers18121964

**Published:** 2026-06-17

**Authors:** Ana Karina Zambrano, Rafael Tamayo-Trujillo, Patricia Guevara-Ramírez, Elius Paz-Cruz, Viviana A. Ruiz-Pozo, Santiago Cadena-Ullauri, Alejandro Cabrera-Andrade, Luis Israel Llerena Béjar

**Affiliations:** 1 Universidad UTE, Facultad de Ciencias de la Salud Eugenio Espejo, Centro de Investigación Genética y Genómica, Quito 170129, Ecuador; 2 Carrera de Enfermería, Facultad de Ciencias de la Salud, Universidad de las Américas, Quito 170125, Ecuador; 3 Grupo de Bio-Quimioinformática, Universidad de las Américas, Quito 170125, Ecuador; 4 Private Practice in Gynecology and Breast Health, Quito 170519, Ecuador

**Keywords:** breast cancer, genetics, genomics, ancestry, Latin America

## Abstract

**Background/Objectives**: Breast cancer (BC) is the most frequently diagnosed malignancy among women worldwide, yet tumor genomic data from Latin American and admixed populations remain limited. This gap affects the interpretation of cancer-associated variants and may limit the representation of these populations in genomic research. This study aimed to characterize the genomic landscape of breast tumors from Ecuadorian women and contextualize variant interpretation through genetic ancestry analysis. **Methods**: Breast tissue samples from 23 Ecuadorian mestizo women with suspected malignancy were analyzed. After histopathological confirmation, 21 breast tumors were included in the final cohort. Targeted next-generation sequencing was performed using a 94-gene cancer panel. Variants were annotated and classified according to clinical significance. Because matched normal DNA was unavailable, variant allele frequency was used as an exploratory approach to infer putative somatic, putative germline, indeterminate, or putative homozygous germline profiles. Putative germline and indeterminate variants were interpreted using ACMG/AMP criteria, whereas putative somatic variants were evaluated using the AMP/ASCO/CAP framework. Genetic ancestry was inferred using ancestry-informative insertion/deletion markers. **Results***:* Seventy-two unique variants across 40 genes were identified, including 14 pathogenic, 10 likely pathogenic, and 48 variants of uncertain significance. Pathogenic and likely pathogenic alterations were detected in clinically relevant genes, including *TP53*, *BRCA2*, *PTEN*, *CDH1*, *RAD51C*, *MSH6*, *NF1*, *CYLD*, and *SDHB*. Variants of uncertain significance represented 66.7% of all detected variants and affected several cancer-associated and DNA repair genes. The cohort showed a trihybrid ancestry profile, with predominant Native American ancestry, followed by European and African contributions. **Conclusions**: This study expands BC genomic data from an underrepresented Ecuadorian cohort and highlights the coexistence of clinically relevant alterations with a high burden of variants of uncertain significance. The findings underscore the limitations of tumor-only sequencing and current variant interpretation frameworks in admixed populations. Larger studies integrating matched normal DNA, ancestry-informed analyses, functional validation, and broader sequencing strategies could help improve variant interpretation in Latin American populations.

## Simple Summary

Breast cancer genomic studies remain underrepresented in Latin American and admixed populations, limiting accurate variant interpretation and equitable precision oncology. We analyzed breast tumors from Ecuadorian women using targeted next-generation sequencing and ancestry-informative markers. Clinically relevant variants were identified in cancer-associated genes, including *TP53*, *BRCA2*, *PTEN*, *CDH1*, and *RAD51C*, together with a high proportion of variants of uncertain significance. Because matched normal DNA was unavailable, tumor-detected variants were interpreted using an exploratory variant allele frequency-based approach to infer putative somatic or germline profiles. Ancestry analysis showed a trihybrid pattern with predominant Native American ancestry, followed by European and African contributions. These findings provide new genomic evidence from an underrepresented Ecuadorian cohort and highlight the need for ancestry-informed interpretation, matched germline testing, and functional validation to achieve precision oncology in admixed populations.

## 1. Introduction

Breast cancer (BC) is the most common malignancy in women and is associated with high rates of morbidity and mortality worldwide [1]. Its incidence is increasing, including among women of reproductive age, particularly in low- and middle-income countries, and has been associated with genetic predisposition, environmental exposures, delayed childbearing, reduced breastfeeding rates, and westernized dietary habits [2].

According to the International Agency for Research on Cancer, there were 2,296,840 BC cases and 666,103 BC-related deaths in women worldwide in 2022, while Ecuador reported 3903 cases and 1154 deaths during the same year [3]. In Ecuador, BC is the most frequently diagnosed malignancy in women and remains a major cause of cancer-related mortality [4]. Over time, the disease burden has increased steadily, with rising incidence, increasing mortality, and projected growth in deaths by 2030 [5]. Moreover, late-stage diagnosis remains common, particularly in rural areas, where stage IV disease can account for up to 50% of cases, reflecting limitations in early detection and access to specialized care [6]. This scenario underscores the need to better understand the biological mechanisms underlying disease progression and disparities in clinical outcomes.

BC is a highly heterogeneous disease with molecular subtypes that influence prognosis and treatment, and distant metastasis is the main cause of death in affected women [7]. This heterogeneity is reflected in histopathology and the expression of molecular markers such as estrogen receptor (ER), progesterone receptor (PR), and human epidermal growth factor receptor 2 (HER2). These markers define the main subgroups: luminal A, luminal B, HER2-enriched, and triple-negative BC (TNBC). Moreover, gene expression profiling has identified additional subtypes that, together with histopathology, guide treatment selection and prognosis [8].

Genetic diversity also contributes to BC heterogeneity, involving multiple genes with different penetrance levels [9]. Pathogenic or likely pathogenic gene variants with high and moderate penetrance, including *BRCA1*, *BRCA2*, *TP53*, *NF1*, *PTEN*, *CHEK2*, *PALB2*, *BARD1*, *RAD51C*, and *STK11*, among others, are associated with increased BC risk [10]. In addition, genome-wide association studies in diverse populations, including underrepresented Latin American cohorts, have identified population-specific variants potentially contributing to disease heterogeneity [9]. These variants are involved in pathways such as PI3K-AKT, TNF-α/NF-κB, p53 signaling, and DNA damage response pathways linked to BC development [11].

Recent advances in BC research, including early detection, risk-adjusted screening, artificial intelligence in imaging, and targeted or immune-based therapies, have improved personalized patient management [12]. In this context, next-generation sequencing (NGS) has become a key tool for tumor genomic profiling, enabling the identification of variants involved in cancer initiation and progression and the detection of clinically actionable biomarkers for precision oncology [13]. However, the genomic landscape of BC in Latin American women remains underrepresented, and the reported variant spectrum differs from that of European or Asian cohorts [14].

Therefore, this study aimed to characterize the genomic landscape of BC tumors in Ecuadorian women using NGS while integrating ancestry analysis to better understand the genetic diversity of this admixed and underrepresented population. This study addresses an important evidence gap by providing genomic and ancestry data from an underrepresented Latin American population, with potential implications for improved diagnosis and personalized treatment strategies.

## 2. Materials and Methods

### 2.1. Study Population

This was a descriptive cross-sectional study. A total of 23 Ecuadorian mestizo women were recruited from a medical center specializing in surgical BC treatment in Quito, Ecuador, between 2024 and 2025. Patients were selected based on clinical evaluation and imaging findings suggestive of breast malignancy and underwent surgical resection of suspicious breast nodules. Resected tissue samples were collected for genomic analysis prior to the availability of definitive histopathological and immunohistochemical results. Subsequent pathological evaluation confirmed BC in 21 cases, whereas two samples corresponded to non-cancer lesions and were excluded from the final analytical cohort.

All participants provided written informed consent. The study was approved by the Research Ethics Committee for Human Subjects of SEK International University (approval code CEISH-UISEK-P-EO-2024-001).

### 2.2. DNA Extraction and Quantification

Tumor tissue samples were collected in 0.9% sodium chloride solution, stored at 2–8 °C, and immediately transported to the Centro de Investigación Genética y Genómica, Universidad UTE, Quito, Ecuador. Genomic DNA was extracted using the PureLink Genomic DNA Mini Kit (Thermo Fisher Scientific, Waltham, MA, USA) according to the manufacturer’s instructions. DNA concentration was determined using the Qubit Broad Range dsDNA Assay Kit (Thermo Fisher Scientific, Waltham, MA, USA). Samples were normalized to 5 ng/µL in a final volume of 10 µL for downstream library preparation.

### 2.3. Next-Generation Sequencing

Targeted next-generation sequencing was performed using the TruSight Cancer Sequencing Panel (Illumina, San Diego, CA, USA), which covers 94 cancer predisposition genes. Libraries were prepared from normalized DNA samples using the TruSight Rapid Capture protocol (Illumina, San Diego, CA, USA) according to the manufacturer’s instructions. Enriched libraries were sequenced on the MiSeq System (Illumina, San Diego, CA, USA) using a v2 300-cycle reagent kit with PhiX as an internal control.

After sequencing, run quality and coverage were reviewed, and only reads with a depth > 20× were retained for analysis. FASTQ files were generated in BaseSpace Sequence Hub (Illumina, San Diego, CA, USA) and processed with DRAGEN Enrichment v3.10.4 (Illumina, San Diego, CA, USA) by alignment to the GRCh38 human reference genome, followed by variant calling for single nucleotide variants and insertions/deletions. Variants not meeting quality thresholds (Q30 scores) were excluded from downstream analysis.

### 2.4. Variant Annotation, VAF-Based Inferred Profile, and Clinical Interpretation

Annotated variants were initially reviewed using the Franklin^®^ platform (Genoox, Tel Aviv, Israel) (https://franklin.genoox.com/clinical-db/home). Variant annotation integrated evidence from public databases, including ClinVar and gnomAD v3.1, the available literature, population frequency data, and multiple in silico prediction tools. Variants were categorized as pathogenic, likely pathogenic, variants of uncertain significance (VUS), likely benign, or benign. Only pathogenic, likely pathogenic, and VUS variants were included in the final analysis, whereas likely benign and benign variants were excluded.

Because matched normal DNA was not available, tumor-detected variants were further assessed using a VAF-based inference approach for tumor-only sequencing data to provide an exploratory classification of variant origin. Variant allele frequency (VAF) values were extracted from the VCF files and used to assign each variant to one of four interpretative profiles: putative somatic, putative germline, indeterminate, or putative homozygous germline. Variants with VAF ≤ 0.45 were considered more compatible with a putative somatic profile, whereas variants with VAF > 0.45 and <0.55 were considered more compatible with a putative heterozygous germline profile; variants with VAF ≥ 0.55 and <1.00 were classified as indeterminate, and variants with VAF equal to 1.00 were classified as putative homozygous germline, following previously reported VAF-based criteria for tumor-only sequencing data [15]. Variants classified as indeterminate were interpreted cautiously because high VAF values in tumor-only sequencing may reflect germline origin, copy-number imbalance, loss of heterozygosity, tumor purity effects, or clonal enrichment.

For clinical interpretation, ACMG/AMP criteria were retained for variants with putative germline, indeterminate, or putative homozygous germline profiles, particularly when evaluating potential hereditary cancer predisposition. In contrast, variants with a putative somatic profile were evaluated using the AMP/ASCO/CAP framework to assign clinical evidence tiers for somatic variants in cancer through the CancerVar (Wang Genomics Lab, Philadelphia, PA, USA) web program (https://cancervar.wglab.org/) [15,16]. This approach was implemented to avoid applying a single interpretative framework uniformly to all tumor-detected variants and to better distinguish variants potentially related to hereditary predisposition from those more compatible with tumor-acquired events. Given the absence of paired normal DNA, the inferred profiles should be interpreted as exploratory and probabilistic rather than definitive evidence of germline or somatic origin. All analyses were descriptive.

### 2.5. Ancestry Inference

Genetic ancestry was estimated using 46 ancestry-informative insertion/deletion markers (AIMs-InDels), analyzed by capillary electrophoresis and fragment analysis on the 3500 Genetic Analyzer (Applied Biosystems, Waltham, MA, USA), following the protocol described by Zambrano et al. [17].

Individual ancestry proportions were inferred with STRUCTURE v2.3.4 (Pritchard Lab, Stanford, CA, USA) using reference populations from the HGDP-CEPH panel, including African, European, and Native American populations (subset H952). Analyses were performed under an admixture model with 10,000 burn-in steps followed by 10,000 MCMC iterations.

## 3. Results

### 3.1. Cohort Characteristics

Breast tissue samples from 23 patients (C01–C23) were collected and sequenced based on clinical and imaging findings suggestive of malignancy before definitive histopathological and immunohistochemical results were available. Following pathological evaluation, two cases (C20 and C23) were classified as non-cancer and were excluded from the analytical cohort, resulting in a final cohort of 21 breast tumors.

**Figure 1:**
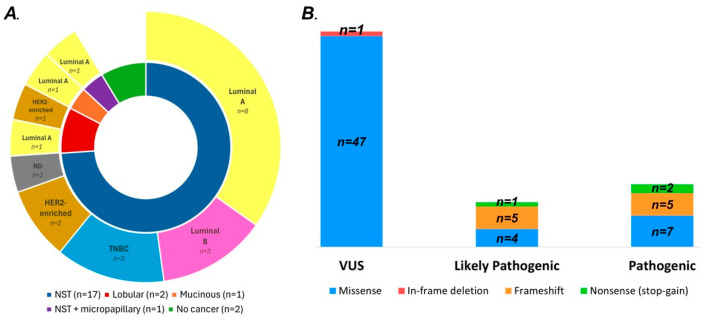
Clinicopathological characteristics and distribution of classified variants in the study cohort. (**A**) Nested donut chart summarizing the final cohort after pathological evaluation. The inner ring shows the WHO histological classification of the 23 sequenced samples, including invasive breast carcinoma of no special type (NST), invasive lobular carcinoma, invasive mucinous carcinoma, invasive breast carcinoma NST with micropapillary component, and two non-cancer cases. The outer ring shows the corresponding immunohistochemical subtypes, including luminal A, luminal B, HER2-enriched, triple-negative BC (TNBC), and one non-determined (ND) case. (**B**) Distribution of variant classification categories stratified by mutation type. Bars represent the number of unique variants classified as variants of uncertain significance (VUS), likely pathogenic, and pathogenic according to the applied clinical interpretation framework, subdivided into missense, frameshift, nonsense (stop-gain), and in-frame deletion alterations.

According to the WHO histological classification, the majority of tumors were classified as invasive breast carcinoma of no special type (NST) (17/21, 81.0%), whereas less frequent histological subtypes included invasive lobular carcinoma (2/21, 9.5%), invasive mucinous carcinoma (1/21, 4.8%), and invasive breast carcinoma NST with micropapillary component (1/21, 4.8%) (Figure 1). To further support the histopathological characterization of the cohort, representative hematoxylin and eosin (H&E)-stained sections of the four histological tumor types identified in this study are provided in Supplementary Figure S1.

Histological grading showed a balanced distribution between low- and intermediate-grade tumors, with grade 1 and grade 2 lesions each representing 38.1% of cases (8/21), while grade 3 tumors accounted for 19.0% (4/21). One additional case exhibited mixed grade 2/3 features (1/21, 4.8%).

Tumor staging information was also incorporated into the baseline clinicopathological characterization of the sequenced breast tissue samples and is provided in Supplementary Table S1. TNM categories were reported separately as primary tumor category (T), regional lymph node category (N), and distant metastasis category (M), together with the clinical/prognostic stage group available from clinical or pathology records. Among the 21 tumors included in the analytical cohort, stage groups ranged from IA to IIIB, and all cases were classified as M0, indicating no documented distant metastasis at diagnosis.

Immunohistochemical subtype classification identified luminal A tumors as the most common subtype (9/21, 42.9%), followed by luminal B (3/21, 14.3%), HER2-enriched (3/21, 14.3%), and triple-negative BC (TNBC) (3/21, 14.3%). One additional case lacked sufficient immunohistochemical information for subtype assignment (1/21, 4.8%) (Figure 1).

### 3.2. Global Variant Landscape

Across the 21 tumors, a total of 72 unique variants affecting 40 distinct genes were identified. According to the applied clinical interpretation framework, 14 variants were classified as pathogenic, 10 as likely pathogenic, and 48 as variants of uncertain significance (VUS). Overall, VUS represented 66.7% (48/72) of all unique variants detected in the cohort (Figure 1).

When grouped by mutation type, most variants corresponded to missense substitutions (58/72, 80.6%), followed by protein-truncating alterations (13/72, 18.1%), including 10 frameshift and 3 nonsense (stop-gain) variants. In addition, one in-frame deletion was identified (1/72, 1.4%).

### 3.3. Genes Harboring Clinically Relevant Pathogenic and Likely Pathogenic Variants

**Figure 2:**
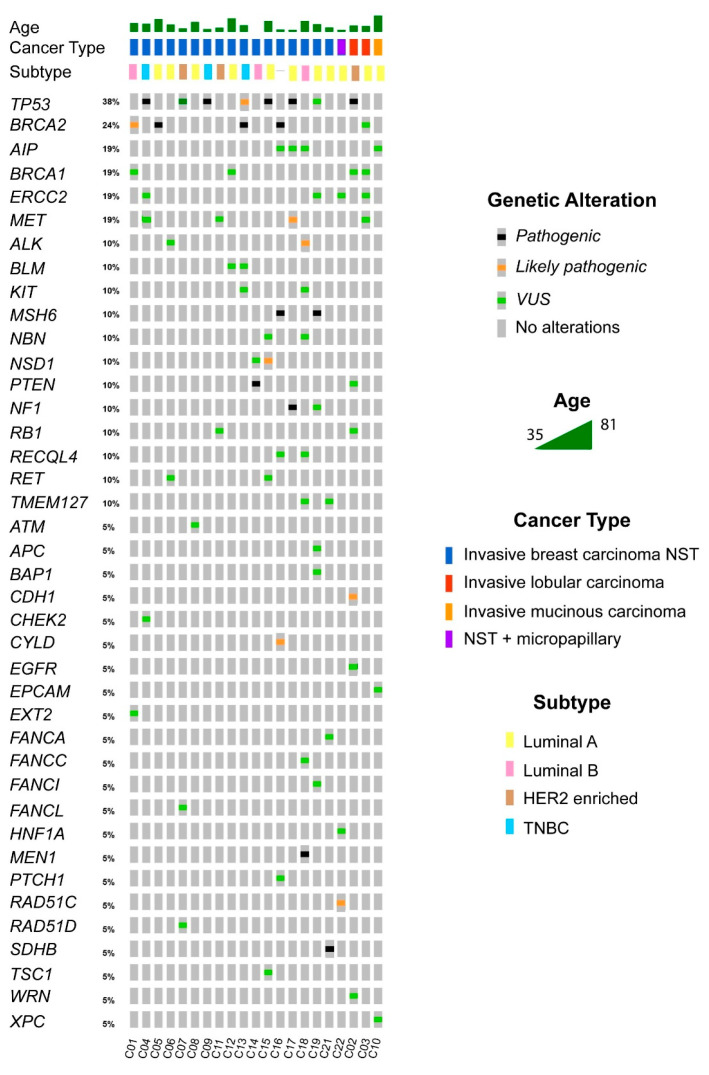
Genomic landscape of breast tumors in the Ecuadorian cohort. Heatmap showing the distribution of genetic alterations across analyzed genes in individual patients. Columns represent individual tumors and rows represent genes harboring at least one detected alteration. Colored squares indicate the presence and classification of variants, including pathogenic, likely pathogenic, and variants of uncertain significance (VUS), while gray indicates no detected alteration. The top annotations show patient age, cancer type, and molecular subtype.

Pathogenic and likely pathogenic variants were identified in several genes with established or potential roles in cancer biology. *TP53* was the most frequently altered gene, harboring 8 unique variants, including both missense and truncating events. *BRCA2* was the second most frequently altered gene, with 4 pathogenic or likely pathogenic unique variants, all corresponding to protein-truncating alterations (Figure 2).

Additional pathogenic and likely pathogenic variants were detected in *CDH1*, *PTEN*, *RAD51C*, *MSH6*, *NF1*, *NSD1*, *CYLD*, *MEN1*, *ALK*, and *SDHB*. These alterations included both protein-truncating variants, observed in genes such as *CDH1*, *PTEN*, *MSH6*, *NF1*, and *CYLD*, and amino acid substitutions identified in *RAD51C*, *NSD1*, *MEN1*, *ALK*, and *SDHB* (Figure 2).

### 3.4. Distribution of Variants of Uncertain Significance (VUS)

Variants of uncertain significance constituted the largest category of alterations in the cohort. A total of 48 unique VUS were distributed across multiple genes, including *BRCA1*, *ATM*, *CHEK2*, *MET*, *RB1*, *RAD51D*, *ERCC2*, *AIP*, *EPCAM*, *WRN*, *BLM*, *KIT*, *NBN*, *TSC1*, *TMEM127*, *FANCA*, *FANCC*, *FANCI*, *BAP1*, *APC*, *HNF1A*, *RET*, *EGFR*, *EXT2*, *XPC*, *PTCH1*, and *RECQL4* (Figure 2).

All *BRCA1* variants identified in this cohort were classified as VUS. Likewise, alterations in genes involved in DNA repair and tumor susceptibility, such as *ATM*, *CHEK2*, *RB1*, and *RAD51D*, were represented exclusively by VUS. In some cases, VUS co-occurred with pathogenic or likely pathogenic variants, whereas in other tumors they represented the only detectable alteration. This pattern underscores the complexity of variant interpretation in multigene sequencing panels and the predominance of uncertain findings within the mutational landscape of this cohort.

A complete list of genomic alterations identified in the cohort is provided in Supplementary Table S2. Variants are presented using HGVS nomenclature together with their molecular consequence, zygosity, available rsID, variant allele frequency (VAF), VAF-based inferred profile, and the corresponding clinical interpretation framework, including ACMG/AMP criteria for variants with putative germline, indeterminate, or putative homozygous germline profiles, and AMP/ASCO/CAP tiers for variants with a putative somatic profile.

### 3.5. Ancestry

**Figure 3:**
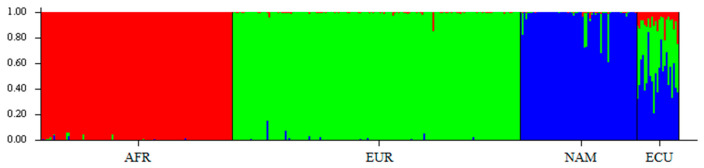
Ancestry composition of the cohort. The bar plot illustrates the ancestral composition of both the analyzed and reference populations, with African ancestry shown in red, European ancestry in light green, and Native American ancestry in blue. The Ecuadorian cohort is displayed as a distinct cluster at the end, characterized by a combination of these colors, reflecting its admixed genetic structure resulting from contributions from these ancestral populations.

Ancestral proportions were analyzed to contextualize the genomic landscape of the cohort. The analysis revealed a trihybrid admixture profile. On average, the cohort exhibited a majority Native American ancestry of 50.9%, followed by a substantial European contribution of 40.1%, and a minor African component of 9.0% (Figure 3).

## 4. Discussion

This study provides a comprehensive description of genetic variants identified in a cohort of BC tumors, highlighting the coexistence of alterations in genes with well-established roles in tumorigenesis and a substantial proportion of variants with uncertain or limited evidence. Given the descriptive nature of this analysis, no assumptions were made regarding the hierarchical contribution of individual variants; however, particular attention was given to genes with previously reported relevance in BC biology.

Among these, several pathogenic and likely pathogenic variants were identified in genes with recognized roles in BC development, including *TP53*, *BRCA2*, *PTEN*, *CDH1*, and *RAD51C* [12]. These genes are involved in key cellular processes such as DNA damage response, homologous recombination repair, cell cycle regulation, and maintenance of epithelial integrity [10]. Alterations in these pathways have been consistently associated with genomic instability and tumor progression. Notably, *TP53*, one of the most frequently mutated genes in human cancer [18], was identified in a considerable proportion of cases, supporting its central role in breast tumor biology.

The *TP53* gene encodes the p53 protein, a central transcription factor in tumor suppression that regulates cellular responses to DNA damage, cell cycle progression, and apoptosis. It is the most frequently mutated gene in human cancers, with most alterations occurring within the DNA-binding domain (residues 102–292), thereby impairing its transcriptional activity [18]. Missense mutations, which predominate, may disrupt DNA binding or destabilize protein structure, leading to loss of function; however, they exhibit marked functional heterogeneity, as some variants exert dominant-negative effects or acquire gain-of-function properties [19]. In BC, these alterations may compromise the activation of key downstream effectors such as p21, BAX, PUMA, and GADD45, thereby affecting cell cycle arrest, apoptosis, and DNA repair, ultimately contributing to genomic instability and tumor progression [19]. In addition, gain-of-function variants have been associated with processes such as proliferation, epithelial–mesenchymal transition, angiogenesis, and immune evasion, while impaired apoptotic responses may reduce the efficacy of DNA-damaging therapies [20].

In our cohort, *TP53* variants were identified in 38.1% (8/21) of cases, a frequency consistent with previous reports in BC [21]. Most of these alterations (7/8) corresponded to clinically relevant pathogenic or likely pathogenic variants according to the applied interpretation framework and included well-characterized missense variants such as p.Arg175His and p.Tyr220Cys, as well as truncating variants resulting from frameshift events. These alterations were predominantly located within the DNA-binding domain, supporting their potential impact on p53 function [18]. In contrast, a single variant of uncertain significance (p.Val31Phe) was identified outside this domain, and its interpretation should be approached with caution given the limited available evidence regarding its functional and clinical relevance.

When stratified by molecular subtype, *TP53* variants were more frequently observed in triple-negative (3/3) and HER2-enriched tumors (2/3) compared to luminal subtypes (3/12), consistent with previous studies linking *TP53* alterations to more aggressive BC phenotypes [20]. Notably, triple-negative tumors exhibited both missense and truncating variants, whereas HER2-enriched tumors were predominantly characterized by missense alterations, and luminal tumors showed a relatively higher proportion of truncating variants. Although the sample size is limited, this distribution suggests potential differences in the mutational spectrum of *TP53* across molecular subtypes [20,21]. Overall, these findings reinforce the importance of considering not only the presence of *TP53* alterations but also their structural context and functional classification when interpreting their potential role in tumor biology.

The *BRCA2* gene plays a critical role in maintaining genomic stability through its involvement in homologous recombination repair of DNA double-strand breaks. Loss-of-function alterations in *BRCA2* impair this high-fidelity repair mechanism, leading to the accumulation of genomic instability, a hallmark of cancer [22]. In BC, pathogenic variants in *BRCA2* are well established in the context of hereditary predisposition; however, their contribution to tumorigenesis depends on the presence of functionally inactivating alterations that compromise protein activity [10]. In our cohort, several truncating *BRCA2* variants, including frameshift and nonsense alterations, were identified and interpreted as clinically relevant pathogenic or likely pathogenic variants according to the applied framework, supporting their potential impact on homologous recombination deficiency [23]. These alterations are expected to disrupt protein function and may contribute to tumor development through defective DNA repair mechanisms [23]. In contrast, additional variants classified as of uncertain significance were also observed, and their biological relevance remains unclear. These findings highlight the importance of distinguishing between clearly inactivating alterations and variants with limited evidence when interpreting the role of *BRCA2* in tumor biology.

The *CDH1* gene encodes E-cadherin, a transmembrane protein essential for epithelial cell–cell adhesion and the maintenance of tissue architecture. Loss-of-function alterations in *CDH1* disrupt cellular adhesion and have been strongly associated with the development of lobular breast carcinoma, where they are considered characteristic molecular events [24]. In this study, a likely pathogenic truncating variant in *CDH1* was identified in a case of lobular carcinoma, consistent with the established role of this gene in this histological subtype. However, not all lobular tumors in the cohort harbored detectable *CDH1* alterations, which may reflect alternative mechanisms of gene inactivation, including structural variants or epigenetic silencing, as well as the inherent molecular heterogeneity of BC [25]. These observations underscore the relevance of *CDH1* in lobular tumor biology while also highlighting that its alteration is not universally observed in all cases.

It is important to note that, although genes such as *BRCA1* and *BRCA2* are widely recognized for their association with hereditary BC susceptibility, their role as tumor drivers depends on the presence of functionally relevant alterations leading to loss of protein activity [10,22,26]. In the context of tumor-only sequencing, however, *BRCA1/2* variants should not be assumed to represent hereditary predisposition without matched normal or orthogonal germline confirmation. Therefore, variants classified as of uncertain significance in these genes should be interpreted with caution, as their biological impact cannot be established based solely on sequence data [26,27]. Similarly, variants identified in genes involved in DNA repair and tumor suppression, including *ATM*, *CHEK2*, *RB1*, and *RAD51D*, may contribute to tumorigenesis depending on the molecular context, although their precise role remains to be clarified in cases lacking definitive pathogenic classification [10,12].

In contrast, a considerable proportion of the detected variants corresponded to genes with limited or unclear evidence regarding their direct involvement in BC, including *ERCC2*, *AIP*, *EPCAM*, *WRN*, and others [10,12,28]. Most of these alterations were classified as variants of uncertain significance, reflecting the current limitations in variant interpretation and the challenges associated with distinguishing biologically relevant events from background genomic variation. Notably, a subset of tumors in our cohort lacked pathogenic alterations in established BC driver genes and instead harbored only VUS in genes with emerging, context-dependent, or still uncertain relevance in breast tumorigenesis. These cases are of particular interest, as they may reflect alternative molecular routes of tumor development that remain insufficiently characterized [28].

One representative example was case C06, corresponding to an invasive breast carcinoma with a luminal A profile, in which two heterozygous VUS were identified in *RET* (p.Val899Gly) and *ALK* (p.Ile1461Leu). Both genes encode receptor tyrosine kinases involved in key oncogenic pathways regulating proliferation, survival, and cellular signaling [29]. In BC, *RET* has been mainly linked to hormone receptor-positive tumors, which is consistent with the luminal A phenotype observed in this case. Its overexpression and activation have been associated with tumor progression, resistance to endocrine therapy, and metastasis through interaction with the estrogen receptor pathway and activation of downstream signaling cascades such as MAPK and PI3K/AKT [30]. In contrast, *ALK* alterations in breast tumors have been more commonly described in aggressive phenotypes through gene fusions, amplifications, or aberrant expression, which differs from the less aggressive luminal A presentation observed in this tumor [31,32]. Although the functional relevance of these variants remains uncertain, their occurrence in a tumor lacking canonical driver alterations supports the possibility of alternative context-dependent mechanisms in tumor development.

Another illustrative case was case C08, diagnosed as an invasive breast carcinoma with a luminal A profile. Genomic analysis identified a heterozygous VUS in *ATM* (p.Ser378Gly). The *ATM* gene encodes a serine/threonine kinase that plays a central role in the DNA damage response, and truncating pathogenic variants are recognized as conferring moderate susceptibility to BC [33]. In contrast, the clinical relevance of many *ATM* missense variants remains uncertain, particularly because only a limited subset affecting critical functional domains has been consistently associated with increased cancer risk [33]. The p.Ser378Gly variant identified in this case is located in the N-terminal region, outside the major catalytic domains most frequently linked to pathogenicity [34]. Therefore, its biological contribution may be limited; however, definitive interpretation requires dedicated functional validation and further clinical evidence.

A further example was case C10, corresponding to an invasive mucinous breast carcinoma with a luminal A profile, in which only VUS were identified in *AIP* (p.Glu24Gln), *EPCAM* (p.Ala71Val), and *XPC* (p.Ala269Gly). These genes participate in distinct cellular processes relevant to tumor biology, including growth regulation, epithelial integrity, and DNA repair [35]. Although *AIP* has not been consistently associated with BC, altered expression has been reported in other tumor types [36]. Likewise, *EPCAM* overexpression has been described in several epithelial malignancies [37], whereas variation in *XPC*, a nucleotide excision repair gene, has been linked to cancer susceptibility in some settings [38]. In the absence of canonical driver alterations, the coexistence of variants affecting multiple biological pathways may reflect combinatorial or low-penetrance mechanisms that deserve further functional investigation.

In addition to the tumors discussed above, two sequenced samples excluded from the final analytical cohort after histopathological evaluation also merit consideration, as both lacked evidence of malignancy yet harbored variants in genes related to cancer-associated pathways. Case C20, in which no neoplastic infiltration was identified, carried VUS in *BLM* (p.Asn1112His) and *MET* (p.Arg591Trp). *BLM* encodes a RecQ helicase involved in DNA repair and maintenance of genomic stability [39], whereas *MET* is a receptor tyrosine kinase associated with cell proliferation, invasion, and survival signaling [40]. Likewise, case C23, also classified as non-neoplastic, harbored VUS in *RET* (p.Tyr900Ser) and *ATM* (p.Gln368Glu), two genes previously discussed in the context of BC biology. Although these findings do not support a diagnosis of BC, they illustrate that alterations in cancer-related genes may also be detected in benign breast lesions or non-neoplastic tissue. From a clinical standpoint, such results should not prompt oncologic treatment in the absence of pathological evidence of malignancy; however, they may justify appropriate clinical surveillance, integration with personal and family risk assessment, and periodic reinterpretation as knowledge on variant pathogenicity continues to expand.

The ancestry analysis of our cohort revealed a trihybrid composition characteristic of Latin American populations and consistent with admixture patterns previously reported in women with BC from several countries in the region [41]. This profile was characterized by a predominance of Native American ancestry (50.9%), followed by European (40.1%) and African (9.0%) components. Previous studies suggest that a higher proportion of European ancestry may be associated with increased overall BC risk, whereas Native American ancestry has been linked to a comparatively protective effect in some populations [41]. In this context, the substantial European contribution observed in our cohort may represent one of several factors influencing susceptibility in mestizo populations.

In addition to its potential relationship with susceptibility, ancestry has also been associated with differences in BC subtype distribution across Latin America. Multicenter studies have reported that a higher degree of European ancestry is associated with a lower probability of HER2-overexpressing tumors, whereas greater Native American ancestry has been linked to a higher frequency of this subtype in several regional cohorts [41,42]. In our series, HER2-enriched tumors represented a minority of cases, a pattern that should be interpreted considering the mixed ancestral composition observed in the cohort. However, given the limited sample size, no definitive association between ancestry proportions and molecular subtype distribution can be established.

Beyond these clinical observations, the ancestral background of the cohort is also highly relevant for genomic interpretation, particularly because most reference databases and pathogenicity frameworks have been developed predominantly from European-ancestry populations. Consequently, variants identified in admixed Latin American individuals are more frequently classified as variants of uncertain significance due to limited population-specific evidence. Accordingly, the high proportion of VUS observed in our cohort may be influenced, at least in part, by the limited availability of genomic data from populations with similar ancestral composition. It is important to note, however, that Eurocentric database bias is not the sole driver of the high VUS rate; the systemic lack of functional experimental validation for a large number of germline variants is an equally critical factor. In this context, specific VUS detected in established BC susceptibility genes in our cohort—including *BRCA1*, *ATM*, *CHEK2*, and *RAD51D*—that are predicted to be potentially deleterious by multiple in silico tools represent priority candidates for future in vitro functional validation. Such validation studies could contribute a population-specific spectrum of actionable pathogenic variants relevant to hereditary BC susceptibility in the Ecuadorian demographic.

This study has several limitations, including the limited sample size, single-center recruitment, and the absence of functional validation for variants of uncertain significance. In addition, because matched normal tissue was not available, variants detected in tumor samples could not be definitively distinguished as somatic or germline in origin. The descriptive design also limits causal interpretation of the contribution of individual variants to tumor development. Therefore, these findings should be interpreted as exploratory and hypothesis-generating. Furthermore, the reliance on a targeted 94-gene panel represents an important methodological constraint. While this approach is practical for clinical diagnostics, it precludes the identification of non-coding variants, structural rearrangements, or novel regional drivers that may be unique to admixed Latin American populations. Future studies employing whole-exome sequencing (WES) or whole-genome sequencing (WGS) would provide a more comprehensive and unbiased characterization of the genomic landscape of BC in this underrepresented population. It is also important to note that ACMG/AMP criteria were applied only to variants inferred as putative germline or when germline origin could not be excluded; while variants inferred as putative somatic were classified according to the AMP/ASCO/CAP framework using Tier categories based on potential clinical significance. Additionally, the high proportion of VUS observed in this cohort reflects not only the Eurocentric bias of public variant databases but also the systemic lack of functional experimental validation for a large number of germline variants. Priority VUS in established BC susceptibility genes (including *BRCA1*, *ATM*, *CHEK2*, and *RAD51D*) predicted as potentially deleterious by multiple in silico tools represent key candidates for future in vitro functional validation, which could contribute a population-specific spectrum of actionable variants for the Ecuadorian demographic.

## 5. Conclusions

This study provides new genomic evidence from an underrepresented Ecuadorian population and reveals the coexistence of pathogenic alterations with a high burden of variants of uncertain significance. The findings highlight persistent limitations in current variant interpretation frameworks for admixed populations and emphasize the value of ancestry-informed approaches. The high proportion of VUS observed reflects both the Eurocentric composition of public reference databases and the systemic lack of functional experimental data for a large number of germline variants, underscoring the need for targeted in vitro validation of priority candidates in this population. It is important to note that the findings of this study should be interpreted as exploratory and hypothesis-generating; direct clinical actionability should not be inferred without further validation. Future studies integrating prospective clinical follow-up, matched germline controls, and unbiased sequencing strategies such as WES or WGS will be essential to bridge the translational gap between genomic characterization and precision oncology outcomes in admixed Ecuadorian and Latin American populations.

## Data Availability

All data generated or analyzed during this study are included in this published article. Additional de-identified data are available from the corresponding author on reasonable request.

## Supplementary Materials

The following supporting information can be downloaded at: https://www.mdpi.com/article/10.3390/cancers18121964/s1, Table S1: Baseline clinicopathological characteristics and TNM staging of the sequenced breast tissue samples. Table S2: Tumor-detected variants, VAF-based inferred profiles, and clinical interpretation framework. Figure S1: Representative histological features of breast tumor types identified in the Ecuadorian cohort according to the WHO Classification of Breast Tumors. Representative hematoxylin and eosin (H&E)-stained sections showing the four histological tumor types identified in the study cohort. (A): Invasive breast carcinoma of no special type. (B): Invasive breast carcinoma of no special type with micropapillary component. (C): Invasive lobular carcinoma. (D): Invasive mucinous carcinoma. These images provide morphological context for the histopathological classification used in the cohort characterization and complement the genomic findings reported in the manuscript.
