# Supplementary material for: Genomic Profiling of Breast Cancer in an Ecuadorian Cohort Reveals Clinically Relevant Variants and Ancestry-Related Interpretation Challenges
Source: Cancers (Basel). 2026 Jun 17;18(12):1964. doi: 10.3390/cancers18121964 (PMC13296644; doi:10.3390/cancers18121964)
Supplement: Supplementary file 1 [file cancers-18-01964-s001.zip › cancers-4332436-supplementary.pdf]

Supplementary Table S1. Baseline clinicopathological characteristics and TNM staging of the sequenced breast tissue samples.

ID	WHO Classification	IHC subtype	Primary tumor category (T)	Regional lymph node category (N)	Distant metastasis category (M)	Stage group[1]
C01	NST	Luminal B	T2	N2	M0	IIIB
C02	Lobular	HER2-enriched	T1c	N1	M0	IIA
C03	Lobular	Luminal A	T2	N0	M0	IIA
C04	NST	TNBC	T2	N0	M0	IIA
C05	NST	Luminal A	T1c	N0	M0	IA
C06	NST	Luminal A	T2	N0	M0	IIA
C07	NST	HER2-enriched	T2	N1	M0	IIB
C08	NST	Luminal A	T1c	N0	M0	IA
C09	NST	TNBC	T2	N1	M0	IIB
C10	Mucinous	Luminal A	T2	N0	M0	IIA
C11	NST	HER2-enriched	T2	N0	M0	IIA
C12	NST	Luminal A	T2	N0	M0	IIA
C13	NST	TNBC	T2	N0	M0	IIA
C14	NST	Luminal B	T2	N0	M0	IIA
C15	NST	Luminal A	T1	N0	M0	IA
C16	NST	Not determined	T1c	N0	M0	IA
C17	NST	Luminal A	T2	N0	M0	IIA
C18	NST	Luminal B	T2	N0	M0	IIA
C19	NST	Luminal A	T2	N0	M0	IIA
C20	No cancer[2]	NA	NA	NA	NA	NA
C21	NST	Luminal A	T3	N0	M0	IIB
C22	NST with micropapillary component	Luminal A	T1c	N0	M0	IIA
C23	No cancer[2]	NA	NA	NA	NA	NA

[1] Stage group corresponds to the clinical/prognostic stage reported in the clinical or pathology records and may incorporate TNM categories, histological grade, and ER, PR, and HER2 status. TNM categories are shown separately as T, N, and M.

[2] C20 and C23 were initially sequenced based on clinical and imaging findings suggestive of malignancy but were classified as non-cancer after definitive histopathological evaluation and excluded from the analytical tumor cohort.

Abbreviations: WHO, World Health Organization; TNM, tumor–node–metastasis; NST, invasive breast carcinoma of no special type; IHC, immunohistochemistry; TNBC, triple-negative breast cancer; HER2, human epidermal growth factor receptor 2; ER, estrogen receptor; PR, progesterone receptor; NA, not applicable.

Supplementary Table S2. Tumor-detected variants, VAF-based inferred profiles, and clinical interpretation framework.

Case ID	Gene	Transcript	HGVSC	HGVSP	Clinical significance	Zygosity	SNP ID	VAF	VAF-based inferred profile	ACMG/AMP criteria or AMP/ASCO/CAP TIER
C01	*BRCA1*	NM_007294.4	c.957C>A	p.Asn319Lys	VUS	Heterozygous	Not reported	0.540	Putative germline	PM2
	BRCA2	NM_000059.4	c.9045dup	p.Ser3016Ilefs*2	Likely Pathogenic	Heterozygous	Not reported	0.719	Indeterminate	PVS1, PM2
	EXT2	NM_207122.2	c.919G>A	p.Asp307Asn	VUS	Heterozygous	rs200631641	0.250	Putative somatic	TIER III
C02	*RB1*	NM_000321.3	c.764G>A	p.Arg255Gln	VUS	Heterozygous	rs746954896	0.402	Putative somatic	TIER II
	TP53	NM_000546.6	c.524G>A	p.Arg175His	Pathogenic	Heterozygous	rs28934578	0.104	Putative somatic	TIER I
	CDH1	NM_004360.5	c.2181_2182dup	p.Leu728Serfs*43	Likely Pathogenic	Heterozygous	Not reported	0.147	Putative somatic	TIER III
	PTEN	NM_000314.8	c.1202C>A	p.Thr401Lys	VUS	Heterozygous	rs1860740005	0.042	Putative somatic	TIER III
	BRCA1	NM_007294.4	c.3230G>A	p.Gly1077Glu	VUS	Heterozygous	rs1567792424	0.075	Putative somatic	TIER III
	EGFR	NM_005228.5	c.2191T>G	p.Trp731Gly	VUS	Heterozygous	Not reported	0.040	Putative somatic	TIER III
	WRN	NM_000553.6	c.1652C>T	p.Pro551Leu	VUS	Heterozygous	rs781107893	0.071	Putative somatic	TIER III
C03	*MET*	NM_000245.4	c.3703T>G	p.Tyr1235Asp	Likely Pathogenic	Heterozygous	rs1057519824	0.042	Putative somatic	TIER III
	BRCA1	NM_007294.4	c.3230G>A	p.Gly1077Glu	VUS	Heterozygous	rs1567792424	0.400	Putative somatic	TIER III
	ERCC2	NM_000400.4	c.410T>G	p.Leu137Arg	VUS	Heterozygous	Not reported	0.100	Putative somatic	TIER III
	BRCA2	NM_000059.4	c.746C>T	p.Ser249Phe	VUS	Heterozygous	rs2137461787	0.159	Putative somatic	TIER III

C04	*TP53*	NM_000546.6	c.537T>A	p.His179Gln	Pathogenic	Heterozygous	rs876660821	0.429	Putative somatic	TIER III
	CHEK2	NM_007194.4	c.904G>A	p.Glu302Lys	VUS	Heterozygous	rs587782460	0.542	Putative germline	PM2, PP3
	ERCC2	NM_000400.4	c.1372T>G	p.Ser458Ala	VUS	Heterozygous	Not reported	0.167	Putative somatic	TIER III
	MET	NM_000245.4	c.3703T>G	p.Tyr1235Asp	VUS	Heterozygous	rs1057519824	0.140	Putative somatic	TIER III
C05	*BRCA2*	NM_000059.4	c.1414C>T	p.Gln472*	Pathogenic	Heterozygous	rs80358429	0.053	Putative somatic	TIER III
C06	*ALK*	NM_004304.5	c.4381A>C	p.Ile1461Leu	VUS	Heterozygous	rs1670283	1,000	Putative homozygous germline[1]	PM2, BP4
	RET	NM_020975.6	c.2696T>G	p.Val899Gly	VUS	Heterozygous	rs2132963224	0.042	Putative somatic	TIER III
C07	*FANCL*	NM_018062.4	c.868A>T	p.Ile290Phe	VUS	Heterozygous	rs1685418592	0.555	Indeterminate	PM2, BP1
	RAD51D	NM_002878.4	c.31G>T	p.Gly11Cys	VUS	Heterozygous	rs776471760	0.600	Indeterminate	PM2
	TP53	NM_000546.6	c.659A>G	p.Tyr220Cys	Pathogenic	Heterozygous	rs121912666	0.308	Putative somatic	TIER I
C08	*ATM*	NM_000051.4	c.1132A>G	p.Ser378Gly	VUS	Heterozygous	rs587779811	0.546	Putative germline	PP3, BP4
C09	*TP53*	NM_000546.6	c.797G>A	p.Gly266Glu	Pathogenic	Heterozygous	rs193920774	0.241	Putative somatic	TIER II
C10	*XPC*	NM_004628.5	c.806C>G	p.Ala269Gly	VUS	Heterozygous	rs899696695	0.488	Putative germline	PM2, BP4
	AIP	NM_003977.4	c.70G>C	p.Glu24Gln	VUS	Heterozygous	rs267606568	0.612	Indeterminate	PM2
	EPCAM	NM_002354.3	92C>T	p.Ala71Val	VUS	Heterozygous	rs567291767	0.087	Putative somatic	TIER III
C11	*MET*	NM_000245.4	c.2318C>T	p.Pro773Leu	VUS	Heterozygous	rs771333219	0.500	Putative germline	PM2, BP6

	RB1	NM_000321.3	c.1037A>C	p.Asp346Ala	VUS	Heterozygous	rs1566194403	0.047	Putative somatic	TIER III
C12	*BLM*	NM_000057.4	c.3334A>C	p.Asn1112His	VUS	Heterozygous	rs1352520577	0.500	Putative germline	PM2
	BRCA1	NM_007294.4	c.3823A>G	p.Ile1275Val	VUS	Heterozygous	rs80357280	0.519	Putative germline	PM2, BP6
C13	*BRCA2*	NM_000059.4	c.8363G>A	p.Trp2788*	Pathogenic	Heterozygous	rs80359080	0.804	Indeterminate	PS4, PVS1, PM2, PP5
	TP53	NM_000546.6	c.366_372del	p.Thr123Argfs*45	Likely Pathogenic	Heterozygous	Not reported	0.680	Indeterminate	PVS1, PM2
	BLM	NM_000057.4	c.1888T>C	p.Ser630Pro	VUS	Heterozygous	rs1567042153	0.169	Putative somatic	TIER III
	KIT	NM_000222.3	c.1666C>A	p.Gln556Lys	VUS	Heterozygous	rs1722301014	0.015	Putative somatic	TIER III
C14	*PTEN*	NM_000314.8	c.829dup	p.Thr277Asnfs*21	Pathogenic	Heterozygous	rs1589665640	0.192	Putative somatic	TIER II
	NSD1	NM_022455.5	c.8066A>G	p.Lys2689Arg	VUS	Heterozygous	rs938172284	0.133	Putative somatic	TIER III
C15	*TP53*	NM_000546.6	c.721del	p.Ser241Profs*6	Pathogenic	Heterozygous	rs2073256180	0.216	Putative somatic	TIER II
	NSD1	NM_022455.5	c.6112A>C	p.Thr2038Pro	Likely Pathogenic	Heterozygous	Not reported	0.178	Putative somatic	TIER III
	RET	NM_020975.6	c.966C>A	p.Asp322Glu	VUS	Heterozygous	rs2132720531	0.048	Putative somatic	TIER III
	NBN	NM_002485.5	c.1662T>G	p.Asp554Glu	VUS	Heterozygous	Not reported	0.037	Putative somatic	TIER III
	TSC1	NM_000368.5	c.3316G>A	p.Glu1106Lys	VUS	Heterozygous	rs2131594768	0.184	Putative somatic	TIER III
C16	*PTCH1*	NM_000264.5	c.650A>T	p.Asp217Val	VUS	Heterozygous	Not reported	0.564	Indeterminate	PM2
	RECQL4	NM_004260.4	c.422A>C	p.Lys141Thr	VUS	Heterozygous	rs1178719750	0.560	Indeterminate	PM2
	AIP	NM_003977.4	c.70G>C	p.Glu24Gln	VUS	Heterozygous	rs267606568	0.575	Indeterminate	PM2
	BRCA2	NM_000059.4	c.1813del	p.Ile605Tyrfs*9	Pathogenic	Heterozygous	rs80359306	0.033	Putative somatic	TIER II

	MSH6	NM_000179.3	c.3261del	p.Phe1088Serfs*2	Pathogenic	Heterozygous	rs267608078	0.067	Putative somatic	TIER III
	CYLD	NM_001378743.1	c.1794C>A	p.Tyr598*	Likely Pathogenic	Heterozygous	Not reported	0.038	Putative somatic	TIER III
C17	*AIP*	NM_003977.4	c.154G>A	p.Asp52Asn	VUS	Heterozygous	rs1193307226	0.553	Indeterminate	PM2, PP3
	TP53	NM_000546.6	c.722C>G	p.Ser241Cys	Pathogenic	Heterozygous	rs28934573	0.319	Putative somatic	TIER I
	NF1	NM_001042492.3	c.8059_8060del	p.Ser2687Cysfs*5	Pathogenic	Heterozygous	rs1060500387	0.255	Putative somatic	TIER II
	TP53	NM_000546.6	c.370del	p.Cys124Alafs*46	Likely Pathogenic	Heterozygous	Not reported	0.037	Putative somatic	TIER III
	NF1	NM_001042492.3	c.5896_5921del	p.Ala1966Serfs*2	Likely Pathogenic	Heterozygous	Not reported	0.065	Putative somatic	TIER III
	MET	NM_000245.4	c.3684C>A	p.Asp1228Glu	Likely Pathogenic	Heterozygous	Not reported	0.052	Putative somatic	TIER III
C18	*MEN1*	NM_001370259.2	c.1354C>T	p.Arg452Trp	Pathogenic	Heterozygous	rs863224810	0.422	Putative somatic	TIER III
	RECQL4	NM_004260.4	c.1372C>T	p.Pro458Ser	VUS	Heterozygous	rs761992231	0.693	Indeterminate	PM2
	ALK	NM_004304.5	c.3718T>G	p.Leu1240Val	Likely Pathogenic	Heterozygous	rs863225282	0.023	Putative somatic	TIER II
	AIP	NM_003977.4	c.374C>A	p.Ala125Glu	VUS	Heterozygous	Not reported	0.051	Putative somatic	TIER III
	TMEM127	NM_017849.4	c.480_482del	p.Gln160del	VUS	Heterozygous	rs777748763	0.068	Putative somatic	TIER III
	KIT	NM_000222.3	c.1879C>T	p.Pro627Ser	VUS	Heterozygous	rs1032761406	0.282	Putative somatic	TIER III
	NBN	NM_002485.5	c.1417C>G	p.Gln473Glu	VUS	Heterozygous	rs755805461	0.314	Putative somatic	TIER III
	FANCC	NM_000136.3	c.1330G>A	p.Val444Ile	VUS	Heterozygous	rs1554828492	0.027	Putative somatic	TIER III
C19	*FANCI*	NM_001113378.2	c.3244C>T	p.Pro1082Ser	VUS	Heterozygous	Not reported	0.425	Putative somatic	TIER III

	TP53	NM_000546.6	c.91G>T	p.Val31Phe	VUS	Heterozygous	rs201753350	0.500	Putative germline	PS3, PM2
	MSH6	NM_000179.3	c.3261del	p.Phe1088Serfs*2	Pathogenic	Heterozygous	rs267608078	0.050	Putative somatic	TIER III
	NF1	NM_001042492.3	c.882G>A	p.Met294Ile	VUS	Heterozygous	Not reported	0.027	Putative somatic	TIER III
	ERCC2	NM_000400.4	c.1219G>T	p.Val407Phe	VUS	Heterozygous	Not reported	0.042	Putative somatic	TIER III
	BAP1	NM_004656.4	c.2072T>G	p.Leu691Arg	VUS	Heterozygous	Not reported	0.064	Putative somatic	TIER III
	APC	NM_000038.6	c.7534A>C	p.Ser2512Arg	VUS	Heterozygous	Not reported	0.061	Putative somatic	TIER III
C20	*BLM*	NM_000057.4	c.3334A>C	p.Asn1112His	VUS	Heterozygous	rs1352520577	0.500	Putative germline	PM2
	MET	NM_000245.4	c.1771C>T	p.Arg591Trp	VUS	Heterozygous	rs45602940	0.450	Putative somatic	TIER III
C21	*SDHB*	NM_003000.3	c.269G>A	p.Arg90Gln	Likely Pathogenic	Heterozygous	rs570278423	0.462	Putative germline	PS4, PS3, PM1, PP2, PM2, PM5, PP3, PP5
	FANCA	NM_000135.4	c.3230T>C	p.Met1077Thr	VUS	Heterozygous	rs776603588	0.519	Putative germline	PM2
	TMEM127	NM_017849.4	c.480_482del	p.Gln160del	VUS	Heterozygous	rs777748763	0.060	Putative somatic	TIER III
C22	*HNF1A*	NM_000545.8	c.521C>T	p.Ala174Val	VUS	Heterozygous	rs201934320	0.500	Putative germline	PM2, PP3, PP2
	ERCC2	NM_000400.4	c.514G>A	p.Ala172Thr	VUS	Heterozygous	rs559154781	0.476	Putative germline	PM2, BP4
	RAD51C	NM_058216.3	c.414G>C	p.Leu138Phe	Likely Pathogenic	Heterozygous	rs267606999	0.035	Putative somatic	TIER III
C23	*RET*	NM_020975.6	c.2699A>C	p.Tyr900Ser	VUS	Heterozygous	Not reported	0.028	Putative somatic	TIER III
	ATM	NM_000051.4	c.1102C>G	p.Gln368Glu	VUS	Heterozygous	rs1565378912	0,444	Probably somatic	TIER II

[1] Putative homozygous germline is a VAF-based inferred profile and does not represent confirmed germline zygosity. Definitive germline origin and zygosity require matched normal DNA or orthogonal germline testing. The VAF-based inferred profile was assigned using tumor-only sequencing data and should not be interpreted as definitive evidence of germline or somatic origin

in the absence of matched normal DNA. ACMG/AMP criteria are shown for variants with putative germline, indeterminate, or putative homozygous germline profiles, whereas AMP/ASCO/CAP tiers are shown for variants with a putative somatic profile.

Abbreviations: VAF, variant allele frequency; HGVSc, Human Genome Variation Society coding DNA nomenclature; HGVSp, Human Genome Variation Society protein nomenclature; VUS, variant of uncertain significance; dbSNP, Single Nucleotide Polymorphism Database; ACMG/AMP, American College of Medical Genetics and Genomics/Association for Molecular Pathology; AMP/ASCO/CAP, Association for Molecular Pathology/American Society of Clinical Oncology/College of American Pathologists.

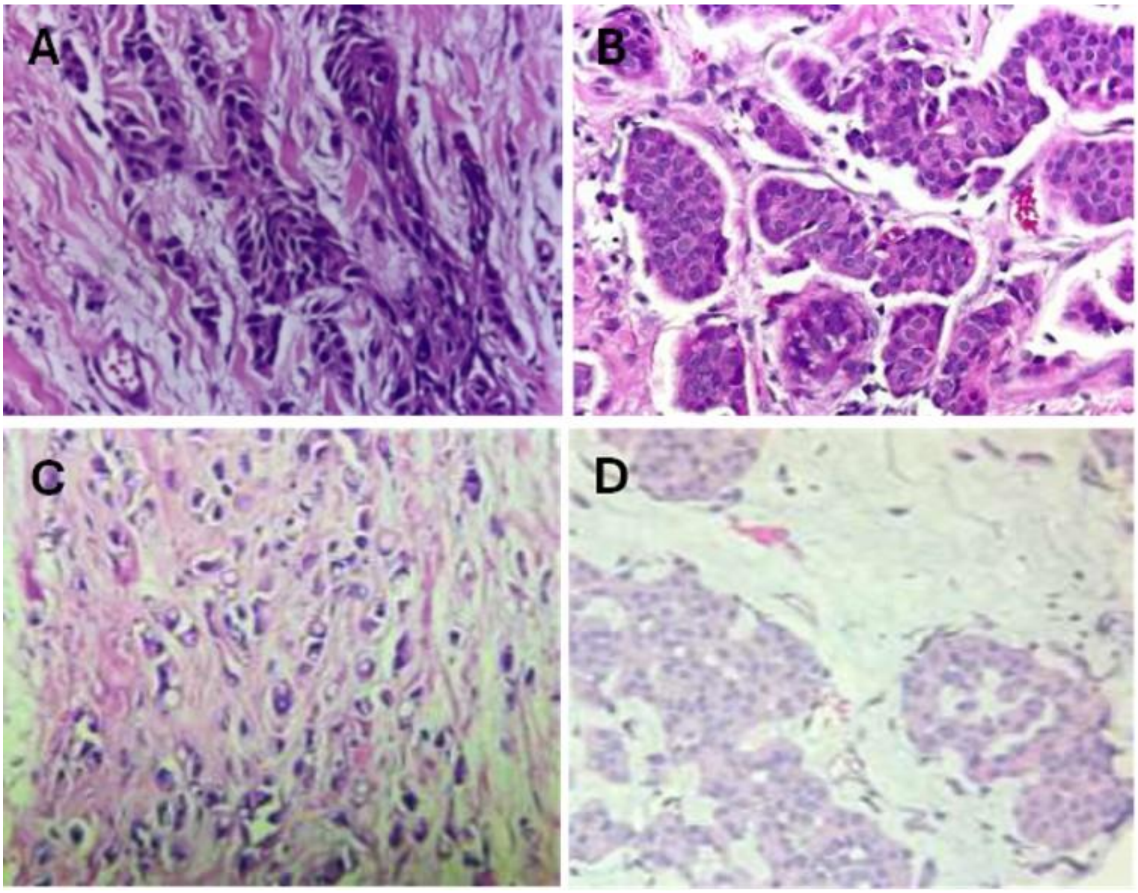

Supplementary Figure S1. Representative histological features of breast tumor types identified in the Ecuadorian cohort according to the WHO Classification of Breast Tumors. Representative hematoxylin and eosin (H&E)-stained sections showing the four histological tumor types identified in the study cohort. (**A**)**:** Invasive breast carcinoma of no special type. (**B**)**:** Invasive breast carcinoma of no special type with micropapillary component. (**C**)**:** Invasive lobular carcinoma. (**D**)**:** Invasive mucinous carcinoma. These images provide morphological context for the histopathological classification used in the cohort characterization and complement the genomic findings reported in the manuscript.
